# Sphingosine 1-Phosphate Counteracts the Effects of Interleukin-1β in Human Chondrocytes

**DOI:** 10.1002/art.37989

**Published:** 2013-07-26

**Authors:** Martin H Stradner, Gerald Gruber, Hannes Angerer, Verena Huber, Daniela Setznagl, Marie-Luise Kremser, Florentine C Moazedi-Fürst, Reinhard Windhager, Winfried B Graninger

**Affiliations:** 1 University of California at San DiegoLa Jolla, California and Medical University of GrazGraz, Austria; 2 Medical University of GrazGraz, Austria; 3 Medical University of ViennaVienna, Austria

## Abstract

**Objective:**

The lipid mediator sphingosine 1-phosphate (S1P) is found in the synovial fluid of osteoarthritis (OA) patients. S1P protects bovine cartilage by counteracting the effects of interleukin-1β (IL-1β). This study was undertaken to examine the interaction of S1P and IL-1β in human OA chondrocytes.

**Methods:**

Human cartilage was obtained from patients undergoing total knee joint replacement. Chondrocytes were cultured in monolayer and treated with IL-1β and S1P. Expression of S1P receptor subtypes and genes involved in cartilage degradation was evaluated using real-time polymerase chain reaction, immunohistochemistry, and Western blotting. S1P signaling was evaluated using inhibitors of S1P receptors and small interfering RNA (siRNA) knockdown of the S1P_2_ receptor. Phosphorylation of MAP kinases and NF-κB in response to IL-1β and S1P was detected by Western blotting.

**Results:**

S1P_2_ was identified as the most prevalent S1P receptor subtype in human OA cartilage and chondrocytes in vitro. S1P reduced expression of inducible nitric oxide synthase (iNOS) in IL-1β–treated chondrocytes. Reduction of ADAMTS-4 and matrix metalloproteinase 13 expression by S1P correlated with S1P_2_ expression. Pharmacologic inhibition of the S1P_2_ receptor, but not the S1P_1_ and S1P_3_ receptors, abrogated the inhibition of iNOS expression. Similar results were observed using siRNA knockdown. S1P signaling inhibited IL-1β–induced phosphorylation of p38 MAPK.

**Conclusion:**

In human chondrocytes, S1P reduces the induction of catabolic genes in the presence of IL-1β. Activation of the S1P_2_ receptor counteracts the detrimental phosphorylation of p38 MAPK by IL-1β.

In osteoarthritis (OA), repeated injury activates chondrocytes to release proinflammatory mediators, cytokines, and matrix-degrading enzymes (1,2). This chronic inflammatory process leads to pathologic joint remodeling and cartilage destruction (1,3). Interleukin-1β (IL-1β) plays a central role in the development and progression of cartilage degradation in OA. Injection of IL-1β into mouse knee joints is sufficient to induce cartilage damage, and elevated levels of IL-1β are found in the synovial fluid of OA patients (4,5).

Upon stimulation with IL-1β, chondrocytes release the matrix-degrading metalloproteases matrix metalloproteinase 1 (MMP-1), MMP-3, MMP-13, and aggrecanase 1 (ADAMTS-4), and inflammatory mediators such as prostaglandins and nitric oxide (NO) (6,7). IL-1β stimulates chondrocytes to release NO by provoking the up-regulation of inducible NO synthase (iNOS; also known as NOS2). NO inhibits the synthesis of proteoglycan and type II collagen (3,8,9). Furthermore, high concentrations of NO induce chondrocyte apoptosis (10). In animal models of OA and rheumatoid arthritis, iNOS-knockout mice exhibit less cartilage degradation compared to their wild-type littermates (11). However, another study did not confirm these results (12). Protein synthesis of iNOS is regulated at the transcriptional level. NF-κB translocation to the nucleus and activation of the MAPK pathways is required for transcription of iNOS, and both processes have been described to occur in response to a variety of stimuli, including IL-1β (13–15).

Physiologic mechanisms that limit the excessive release of NO from human chondrocytes are poorly understood. We have previously reported that the endogenous bioactive sphingolipid sphingosine 1-phosphate (S1P) is able to counteract the effects of IL-1β and diminish the expression of iNOS, MMP-13, and ADAMTS-4 in bovine chondrocytes (16). S1P is generated by sphingosine kinase from the ceramide metabolite sphingosine (17). It is involved in the regulation of vital functions, including cell migration, inflammation, angiogenesis, and wound healing (18–20). S1P exerts its various functions by binding to specific G protein–coupled receptors, of which 5 functionally different isoforms (termed S1P_1–5_) have been identified. We and others have described gene expression of these receptors in bovine, rat, and human chondrocytes (16,21,22). S1P is present in the synovial fluid of OA patients, and synovial tissue is a potential source of S1P (23,24). In human chondrocytes, S1P has been implicated in the regulation of cyclooxygenase 2 and vascular endothelial growth factor (25,26). The current study investigates the effects of S1P on IL-1β signaling and on the expression of iNOS, MMP-13, and ADAMTS-4 in human OA chondrocytes. In addition, we define the receptors and signaling pathways involved in this process.

## MATERIALS AND METHODS

### Reagents

S1P (Sigma-Aldrich) was dissolved in methanol, evaporated, and then resuspended in 0.4% fatty acid–free bovine serum albumin (PAA Laboratories). Recombinant human IL-1β (10 ng/ml; Sigma-Aldrich) was dissolved in water. U0125 (50 μ*M*; Cell Signaling Technology), JTE-013 (10 μ*M*; Tocris Bioscience), PD169316 (30 μ*M*; Sigma-Aldrich), SP600125 (20 μ*M*; Tocris Bioscience), Y-27632 (10 μ*M*; Sigma-Aldrich), pertussis toxin (PTX) (100 ng/ml; Merck Chemicals), and suramin (5 μ*M*; Sigma-Aldrich) were used. High-glucose Dulbecco's modified Eagle's medium (DMEM) with l-glutamine, DMEM/Ham's F-12 with l-glutamine (1:1), fetal bovine serum (FBS), and penicillin/streptomycin solution were purchased at PAA Laboratories. We acquired iNOS antibodies (Cayman Chemical) and antibodies for β-actin (Sigma-Aldrich), as well as antibodies for JNK-1/2/3 and phospho–JNK-1/2/3, ERK-1/2 and phospho–ERK-1/2, p38 MAPK and phospho–p38 MAPK, and NF-κB p65 and phospho–NF-κB p65, and secondary antibodies (all from Cell Signaling Technology). For immunohistochemistry, S1P_1_ antibody (GenWay Biotech), S1P_2_ antibody (Acris Antibodies), S1P_3_ antibody (Cayman Chemical), and a polyclonal swine/anti-goat/anti-mouse/anti-rabbit antibody (Dako) were used.

### Histology

Human cartilage was obtained from patients undergoing total knee joint replacement. Patients gave their informed consent in accordance with the protocol approved by the local ethics committee. Cartilage specimens were extracted from macroscopically intact and damaged areas of the same knee. The specimens were fixed in 3.7% formaldehyde for 24 hours and then embedded in paraffin. For histologic grading, sections of 3 μm were stained with 0.1% Safranin O solution, 0.001% fast green solution, and Weigert's iron hematoxylin solution (27). Sections were graded according to the Osteoarthritis Research Society International (OARSI) histologic grading system (28).

Immunohistochemical analyses were done after pretreatment of the cartilage tissue with proteinase type 24 (for S1P_1_; Sigma-Aldrich) or proteinase K (for S1P_2_ and S1P_3_; Dako) for 3 minutes. Thereafter, the specimens were incubated with primary antibodies (dilution 1:50 for S1P_1_, 1:100 for S1P_2_, and 1:100 for S1P_3_) for 1 hour at room temperature, followed by washing and incubation with secondary antibodies (dilution 1:100) for 30 minutes. Negative controls were incubated with the secondary antibody only. Signals were visualized using a commercially available kit based on 3-amino-9-ethyl-carbazole reaction to streptavidin–peroxidase (Dako).

### Cell culture of human chondrocytes

To obtain primary chondrocytes for monolayer cell culture, cartilage was minced and digested in 0.2% collagenase B (Hoffman-La Roche) for 16 hours. The resulting cell suspension was filtered through a nylon mesh with pores of 70 μm (BD Pharma). Cells were counted and cell viability was tested using trypan blue dye (Sigma-Aldrich). Human chondrocytes were then expanded in monolayer at 37°C, in an atmosphere of 5% CO_2_ and 5% O_2_, in DMEM/Ham's F-12 (1:1) supplemented with 10% FBS and 1% penicillin–streptomycin solution over 3 passages. After the cells had reached 80–90% confluence, serum-free medium was added to the cultures at 24 hours prior to initiation of the experiments.

### Quantitative real-time reverse transcription–polymerase chain reaction (RT-PCR)

For real-time RT-PCR analysis, chondrocytes (from duplicate samples) were treated with 10 ng/ml IL-1β or 100 ng/ml tumor necrosis factor α (TNFα) in combination with 0.1–6 μ*M* S1P or vehicle solution for 3–12 hours. Four independent experiments with chondrocytes derived from 4 different patients were performed. RNA isolation and complementary DNA (cDNA) synthesis were performed as described in an earlier study (29). For amplification, a ready-to-use Master Mix containing SYBR Green (Invitrogen) was used. Primers were purchased at MWG Biotech (primer sequences are available from the corresponding author upon request). The initial amount of cDNA was calculated using ABI Prism sequence detection software (Applied Biosystems) in accordance with the manufacturer's manual, and was based on fixed quantities for the standard. The housekeeping genes GAPDH and hypoxanthine phosphoribosyltransferase 1 served as an internal control, and expression of the target genes was compared to the geometric mean of both housekeeping genes. All samples were run in triplicate.

### Western blot analysis

After 5 minutes or 12 hours of treatment of the chondrocytes in cultures with 10 ng/ml IL-1β in combination with 3 μ*M* S1P or vehicle solution, total protein was extracted using radioimmunoprecipitation assay buffer supplemented with 1% protease inhibitor cocktail (both from Sigma-Aldrich). Four independent experiments using chondrocytes derived from 3 different patients were performed. The protein concentration was measured with a DC Protein assay (Bio-Rad). Proteins were separated by sodium dodecyl sulfate–polyacrylamide gel electrophoresis using a 10% polyacrylamide gel, and then transferred to a nitrocellulose membrane (Bio-Rad). After blocking in 5% skim milk–Tris buffered saline, the membranes were incubated with primary antibodies overnight. Thereafter, the membranes were rinsed in blocking solution and incubated for 1 hour with a secondary antibody conjugated to horseradish peroxidase. Bands were visualized using an acridan-based substrate detection system (ECL Plus; Amersham).

### Analysis of NOS and ADAMTS activity

Chondrocytes (from triplicate samples) from 4 different patients were treated with 10 ng/ml IL-1β in combination with 0.5–6 μ*M* S1P or vehicle solution for 24 hours. Supernatants were analyzed for nitrate concentrations as an indicator of NO release. Griess reaction was performed as described previously (29). The same supernatants were also analyzed for ADAMTS activity using a commercially available enzyme-linked immunosorbent assay (ELISA) (ProteaDetect Sensitive Aggrecanase Activity Assay; ProteaImmun). Briefly, 170 μl supernatant or 5 μl of the provided ADAMTS-4 standards were incubated with 95 μl of 1 μ*M* aggrecan interglobular domain for 6 hours at 37°C. The resulting cleavage fragments were quantified for the ARGSVIL peptide by ELISA. Results were normalized to the total protein concentration in the lysed chondrocytes.

### Small interfering RNA (siRNA) knockdown

The siRNA oligonucleotides were purchased from Microsynth (a full list is available from the corresponding author upon request). For these experiments, siRNA oligonucleotides targeting PTEN and S1P_2_ RNA were used. We used a scrambled siRNA as the negative control. Transfection was carried out using siPORT NeoFX transfection agent (Ambion), in accordance with the manufacturer's recommendations. Forty-eight hours after transfection, chondrocytes were treated for 3 hours with 10 ng/ml IL-1β in combination with 3 μ*M* S1P or vehicle solution. The degree of knockdown was determined by quantitative RT-PCR.

### Statistical analysis

Results are presented as the mean ± SEM. Real-time RT-PCR results are expressed as mean ± SEM percentage over control. Normal distribution of the data was assessed using the Kolmogorov-Smirnov test. Data in all treatment groups were normally distributed. Data were analyzed by two-way analysis of variance followed by post hoc analysis using *t*-tests for Bonferroni correction. Correlations were analyzed by calculating the Pearson's product-moment correlation coefficient or Spearman's rank correlation coefficient for ordinal data (OARSI histologic grades). GraphPad Prism software (version 5) was used for statistical analyses. Differences between groups were considered significant at *P* values less than 0.05.

## RESULTS

**Figure 1:**
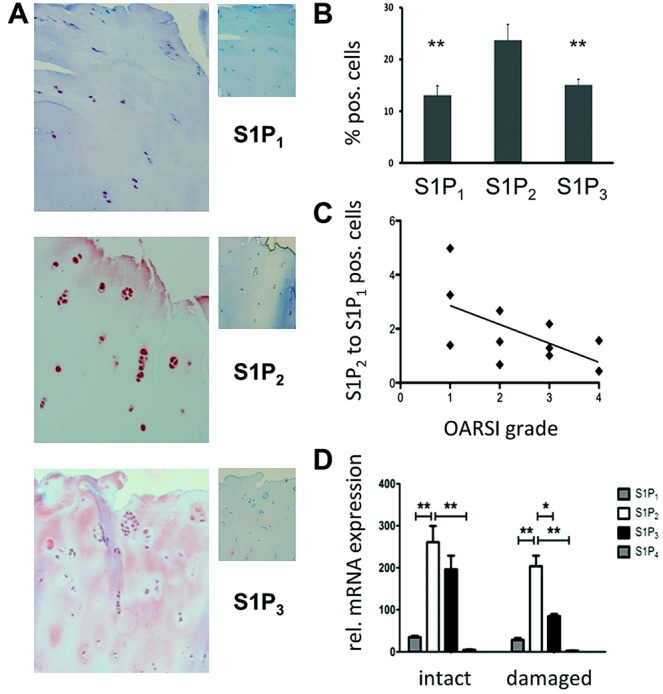
Sphingosine 1-phosphate (S1P) receptors in human osteoarthritis (OA) cartilage. **A,** Immunohistochemical staining for S1P receptors. Larger images (left) are representative of 22 samples from 11 OA patients. Smaller images (right) show the respective negative controls. Original magnification × 200. **B,** Relative frequency of S1P receptors in human OA chondrocytes. Results are the mean ± SEM percentage of receptor-positive cells in 18 OA samples. ∗∗ = *P* < 0.01 versus S1P_2_. **C,** Ratio of S1P_2_-positive cells to S1P_1_-positive cells in relation to Osteoarthritis Research Society International (OARSI) histologic grades in 11 OA samples. **D,** Relative (rel.) expression of S1P receptor mRNA in cultured human OA chondrocytes derived from either intact cartilage or damaged areas of the joint cartilage, as quantified by real-time reverse transcription–polymerase chain reaction (PCR). Results are the mean ± SEM arbitrary units, corrected for PCR efficiency and normalized to values for housekeeping genes, in 6 samples per group. ∗ = *P* < 0.05; ∗∗ = *P* < 0.01.

To investigate the distribution of S1P receptors in OA cartilage ex vivo (n = 22 specimens from 11 patients), we performed immunohistochemical staining for these receptors (Figure 1A). Positive staining for S1P receptors was found in a mean ± SEM 13.2 ± 8.0% of chondrocytes, 23.7 ± 12.6% of chondrocytes, and 15.1 ± 4.4% of chondrocytes for S1P_1_, S1P_2_, and S1P_3_, respectively (Figure 1B). Overall, S1P_2_ was the most prevalent receptor subtype (*P* = 0.003 versus S1P_1_, and *P* = 0.01 versus S1P_3_). Chondrocytes expressing S1P receptors were more abundant in the superficial layer and in chondrocyte clusters.

To assess possible correlations of S1P receptor expression with the extent of cartilage damage, we graded serial sections according to the OARSI cartilage histopathology grading system, resulting in OARSI grades ranging from 1 to 4 (mean ± SEM histologic grade 2 ± 0.2 for all cartilage samples). We observed a nonsignificant trend toward a higher percentage of S1P receptor–positive chondrocytes in samples with higher OA histologic grades. Interestingly, with increasing cartilage damage, more chondrocytes expressed S1P_1_ and fewer expressed S1P_2_. Indeed, the ratio of S1P_2_-expressing chondrocytes to S1P_1_-expressing chondrocytes in the individual samples inversely correlated with the extent of cartilage damage (r = −1, *P* = 0.042) (Figure 1C). We did not find a similar correlation when we compared the ratios of S1P_2_ to S1P_3_ or S1P_3_ to S1P_1_ (results not shown).

We then quantified the level of S1P receptor messenger RNA (mRNA) in cultured chondrocytes derived from macroscopically intact cartilage or from damaged areas of cartilage. Similar to the findings in chondrocytes ex vivo, S1P_2_ was the receptor expressed most abundantly in chondrocytes from both intact and damaged cartilage (Figure 1D). As expected from previous data, we found that S1P_4_ was expressed at a low level and S1P_5_ was not detectable in chondrocytes derived from either intact or damaged cartilage (results not shown).

**Figure 2:**
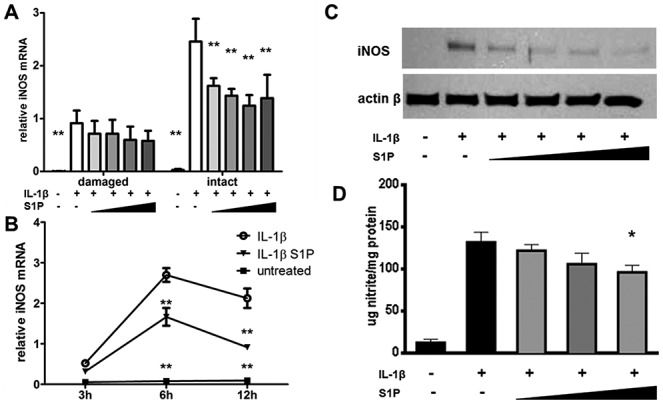
Reduction in inducible nitric oxide synthase (iNOS) expression by sphingosine 1-phosphate (S1P). **A** and **B,** Levels of iNOS mRNA in chondrocytes from either damaged cartilage or intact cartilage (**A**) and in chondrocytes from damaged cartilage over time (**B**) were quantified by real-time reverse transcription–polymerase chain reaction after treatment with 10 ng/ml interleukin-1β (IL-1β) along with vehicle solution or S1P (in **A,** increasing concentrations of 0.1 μ*M*, 0.5 μ*M*, 3 μ*M*, and 6 μ*M* for 3 hours; in **B,** concentration of 3 μ*M* for 3, 6, and 12 hours). Results are the mean ± SEM arbitrary units, normalized to the values for housekeeping genes, in samples from 4 patients (**A**) or 6 patients (**B**). **C,** Western blotting was used to assess iNOS expression, relative to β-actin, in chondrocytes from intact cartilage treated with 10 ng/ml IL-1β and vehicle solution or increasing concentrations of S1P (0.1 μ*M*, 0.5 μ*M*, 3 μ*M*, 6 μ*M*) for 12 hours. Representative results from 1 of 3 samples are shown. **D,** Nitrite accumulation was determined in the supernatants of chondrocytes treated with 10 ng/ml IL-1β and vehicle solution or increasing concentrations of S1P (0.5 μ*M*, 3 μ*M*, 6 μ*M*) for 24 hours. Results are the mean ± SEM levels in chondrocyte cultures derived from 4 samples. ∗ = *P* < 0.05; ∗∗ = *P* < 0.01 versus IL-1β–treated vehicle control.

In bovine chondrocytes, cotreatment of the cells with S1P counteracted the induction of expression of iNOS, ADAMTS-4, and MMP-13 by IL-1β (16). Using these same conditions in human chondrocytes, we observed a prompt and significant decrease in the levels of iNOS mRNA in chondrocytes from intact cartilage after cotreatment with S1P (Figure 2A). In contrast, chondrocytes from damaged cartilage expressed lower levels of iNOS mRNA upon stimulation with IL-1β and did not respond to S1P after 3 hours of cotreatment (Figure 2A). A longer period of incubation of the cells from damaged cartilage restored the inhibitory effect of S1P, which was most pronounced after 12 hours (Figure 2B).

**Figure 3:**
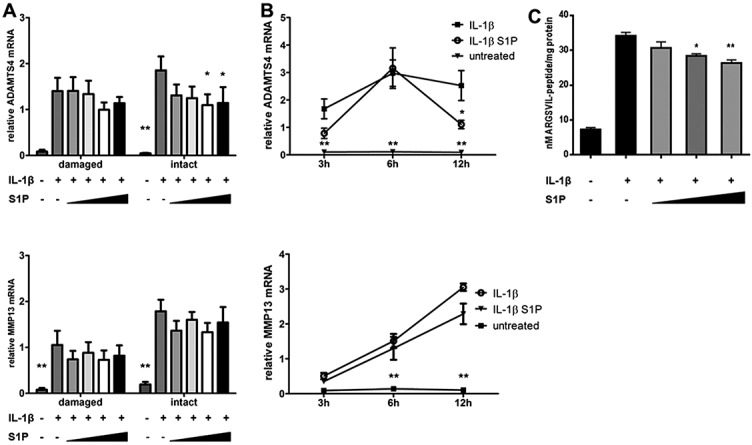
Effect of sphingosine 1-phosphate (S1P) on expression of ADAMTS-4 and matrix metalloproteinase 13 (MMP-13) in cartilage chondrocytes. **A,** Expression of ADAMTS-4 mRNA (upper panel) and MMP-13 mRNA (lower panel) in chondrocytes from either damaged cartilage or intact cartilage was quantified by real-time reverse transcription–polymerase chain reaction (RT-PCR) after 3 hours of treatment with 10 ng/ml interleukin-1β (IL-1β) and vehicle solution or increasing concentrations of S1P (0.1 μ*M*, 0.5 μ*M*, 3 μ*M*, 6 μ*M*). Results are the mean ± SEM arbitrary units, normalized to the values for housekeeping genes, in 3 cartilage samples. **B,** Expression of ADAMTS-4 and MMP-13 mRNA in chondrocytes from damaged cartilage over time was assessed by real-time RT-PCR after treatment with 10 ng/ml IL-1β along with vehicle solution or 3 μ*M* S1P. Results are the mean ± SEM arbitrary units in chondrocyte cultures derived from 6 cartilage samples. **C,** ADAMTS activity was assessed in the supernatants of chondrocytes treated with 10 ng/ml IL-1β along with vehicle solution or increasing concentrations of S1P (0.5 μ*M*, 3 μ*M*, 6 μ*M*) for 24 hours. Results are the mean ± SEM in chondrocyte cultures derived from 4 samples. ∗ = *P* < 0.05; ∗∗ = *P* < 0.01 versus IL-1β–treated vehicle control.

Consistent with the changes in mRNA expression, iNOS protein production and NOS activity in the chondrocytes were found to be induced by stimulation with IL-1β and considerably reduced by cotreatment with S1P (Figures 2C and D). S1P also counteracted the IL-1β–induced expression of ADAMTS-4 in chondrocytes from intact cartilage, but not in chondrocytes from damaged cartilage, after 3 hours of cotreatment (Figure 3A). A significant reduction in ADAMTS-4 expression in chondrocytes from damaged cartilage was observed after 12 hours of cotreatment (Figure 3B). Furthermore, ADAMTS activity in the supernatants of S1P-cotreated chondrocytes from intact cartilage was significantly reduced when compared to that in chondrocytes from vehicle-treated intact cartilage (Figure 3C). The influence of S1P on IL-1β–induced expression of MMP-13 was heterogeneous in the cartilage samples examined; no significant reduction in MMP-13 expression was observed following S1P cotreatment of the cartilage chondrocytes (Figures 3A and B).

We next tested whether the reduction in expression of iNOS and ADAMTS-4 was the result of a specific interaction with IL-1β. Chondrocytes stimulated with TNFα also up-regulated mRNA expression of iNOS, ADAMTS-4, and MMP-13 (results available from the corresponding author upon request). Costimulation with S1P reduced the expression of iNOS in chondrocytes from both intact cartilage and damaged cartilage, whereas expression of ADAMTS-4 and MMP-13 mRNA was diminished in chondrocytes from intact cartilage only.

**Figure 4:**
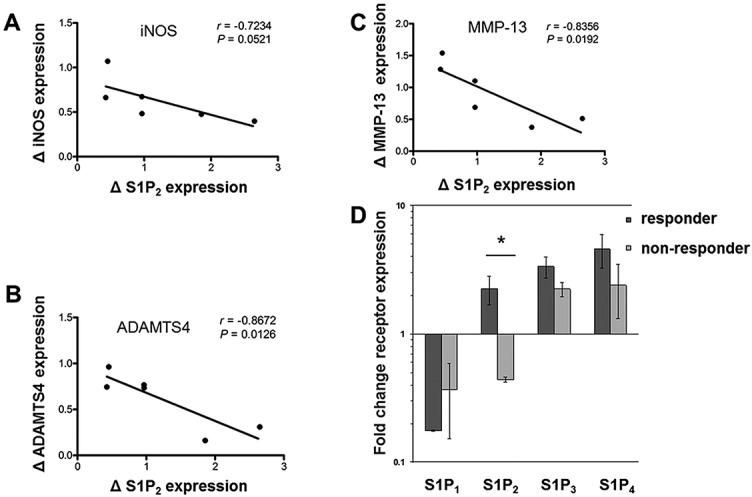
Correlations between interleukin-1β (IL-1β)–induced up-regulation of sphingosine 1-phosphate receptor 2 (S1P_2_) expression in chondrocytes and the optimal inhibitory effect of S1P. **A–C,** The IL-1β–induced change in S1P_2_ receptor expression in chondrocytes from damaged cartilage was assessed for correlations with the suppressed transcription of inducible nitric oxide synthase (iNOS) (**A**), ADAMTS-4 (**B**), and matrix metalloproteinase 13 (MMP-13) (**C**). Chondrocytes derived from damaged cartilage (6 samples) were treated with 10 ng/ml IL-1β and either vehicle solution or 3 μ*M* S1P, and changes in mRNA expression were assessed by real-time reverse transcription–polymerase chain reaction (RT-PCR). Values were normalized to those for housekeeping genes, and correlations were determined using Pearson's correlation coefficient. **D,** Chondrocytes derived from damaged cartilage (3 samples) were treated with 10 ng/ml IL-1β and 3 μ*M* S1P, and inhibition of iNOS expression by S1P was assessed using real-time RT-PCR. Responders were defined as chondrocytes from damaged cartilage that had responded to treatment with 3 μ*M* S1P by showing a reduction in the levels of iNOS mRNA of at least 50% after 12 hours. Heterogeneity in IL-1β regulation of S1P receptor expression in damaged cartilage was observed. Results are the mean ± SEM fold change relative to untreated controls, with values normalized to those for the housekeeping genes. ∗ = *P* < 0.05.

To find an explanation for the delayed and inhomogeneous response of chondrocytes from damaged cartilage to S1P, we tested whether S1P receptor expression was influenced by IL-1β. Chondrocytes from damaged cartilage exhibited lower basal expression of S1P_2_ and S1P_3_ (Figure 1D). After 12 hours of treatment with IL-1β, we observed marked changes in S1P receptor expression. S1P_1_ was down-regulated, whereas S1P_3_ and S1P_4_ were up-regulated, by IL-1β. S1P_2_ showed considerable variation and was up-regulated in some cartilage samples, whereas it was down-regulated in others. Interestingly, this variation correlated with the extent of inhibition of iNOS, ADAMTS-4, and MMP-13 mRNA by S1P (Figures 4A–C). No correlation between the extent of inhibition of expression of either iNOS, ADAMTS-4, or MMP-13 and the expression of S1P_1_, S1P_3_, or S1P_4_ was found.

Furthermore, we analyzed S1P receptor expression in chondrocytes from damaged cartilage in which the response to S1P had been at the same magnitude as that in chondrocytes from intact cartilage (i.e., using a cutoff of 50% reduction in iNOS mRNA expression as the definition of responder) in comparison with chondrocytes from damaged cartilage in which this level of response had not been reached (i.e., nonresponder). We found that regulation of S1P_2_ receptor expression by IL-1β was significantly different between the responder and nonresponder groups (Figure 4D). These results indicate that up-regulation of S1P_2_ by IL-1β was correlated with the optimal response to S1P in chondrocytes from damaged cartilage.

**Figure 5:**
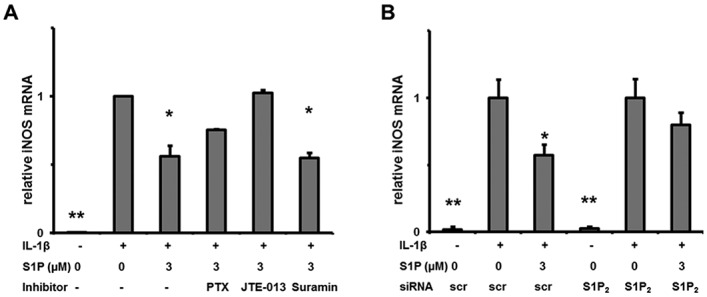
Effect of receptor inhibition on inducible nitric oxide synthase (iNOS) expression. **A,** Chondrocytes were treated with 10 ng/ml interleukin-1β (IL-1β) along with 3 μ*M* sphingosine 1-phosphate (S1P), 100 ng/ml pertussis toxin (PTX) (inhibitor of G_αi_/S1P_1_), 10 μ*M* JTE-013 (inhibitor of S1P_2_), or 5 μ*M* suramin (inhibitor of S1P_3_) for 3 hours. Expression of iNOS mRNA was quantified by real-time reverse transcription–polymerase chain reaction (RT-PCR). **B,** Chondrocytes were transfected with small interfering RNA (siRNA) against S1P_2_ or a scrambled control (scr), and 24 hours after transfection, chondrocytes were treated with 10 ng/ml IL-1β and 3 μ*M* S1P for 3 hours. Expression of iNOS mRNA was quantified by real-time RT-PCR. Results are the mean ± SEM arbitrary units, normalized to the values for the housekeeping genes, in 3 cartilage samples. ∗ = *P* < 0.05; ∗∗ = *P* < 0.01 versus IL-1β–treated vehicle control.

Based on these observations, we wanted to evaluate whether S1P_2_ signaling was needed for the reduction in IL-1β–induced iNOS expression. In chondrocytes incubated with JTE-013, an inhibitor of S1P_2_ receptor signaling, the effect of S1P on iNOS expression was fully reversed (Figure 5A). Neither incubation with suramin, which selectively inhibits S1P_3_, nor incubation with PTX, which is an inhibitor of G_αi_ signaling (required for functional S1P_1_), reversed the effects of S1P (20).

To confirm this result, we utilized siRNA technology for knockdown of S1P_2_. The S1P_2_-knockdown efficacy in primary human chondrocytes was confirmed by the observed reduction in S1P_2_ mRNA expression of 61 ± 7% (mean ± SD) (*P* = 0.012). In chondrocytes transfected with non-sense siRNA, iNOS mRNA expression was significantly reduced (*P* = 0.0341) after treatment with S1P; in cells transfected with S1P_2_ siRNA, the effect of S1P was diminished (*P* = 0.0955) (Figure 5B). We observed similar results in chondrocytes stimulated with TNFα (available from the corresponding author upon request).

We next evaluated the activation of signaling molecules downstream of S1P_2_ (24). However, neither pharmacologic inhibition of Rho-associated protein kinase (ROCK) using Y-27632 nor siRNA-mediated knockdown of PTEN had an impact on S1P signaling (results available from the corresponding author upon request).

**Figure 6:**
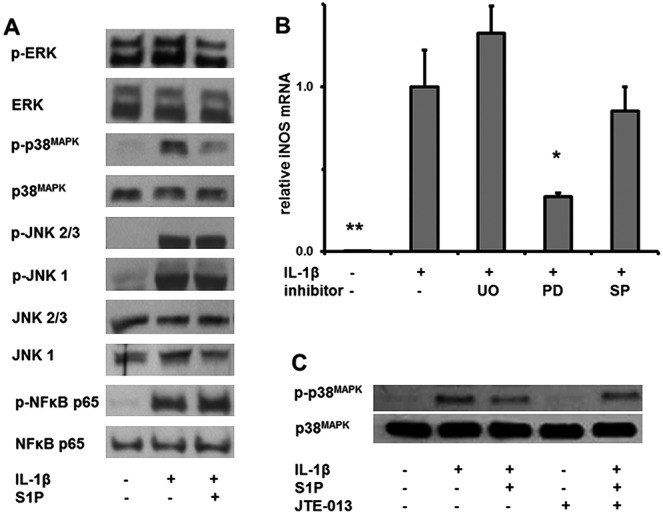
Inhibition of p38 MAPK phosphorylation by sphingosine 1-phosphate (S1P). **A,** Western blotting was used to assess total proteins in chondrocytes isolated 5 minutes after treatment with 3 μ*M* S1P and 10 ng/ml interleukin-1β (IL-1β). The binding of antibodies against phosphorylated (p) and unphosphorylated kinases and transcription factors is visualized. Representative results from 1 of 3 samples are shown. **B,** Chondrocytes were treated with 10 ng/ml IL-1β along with 50 μ*M* U0126 (UO; inhibitor of ERK-1/2), 30 μ*M* PD169316 (PD; inhibitor of p38 MAPK), or 20 μ*M* SP600125 (SP; inhibitor of JNK) for 3 hours. Expression of inducible nitric oxide synthase (iNOS) mRNA was quantified by real-time reverse transcription–polymerase chain reaction. Results are the mean ± SEM arbitrary units, normalized to the values for the housekeeping genes, in 3 samples. ∗ = *P* < 0.05; ∗∗ = *P* < 0.01 versus chondrocytes treated with IL-1β alone. **C,** Phosphorylated and total p38 MAPK from total proteins was assessed by Western blotting in chondrocytes isolated 5 minutes after treatment with 3 μ*M* S1P, 10 ng/ml IL-1β, and 10 μ*M* JTE-013. Representative results from 1 of 3 samples are shown.

As IL-1β appears to induce the expression of iNOS independently via the NF-κB and MAPK pathways, we examined the phosphorylation of NF-κB p65, ERK-1/2, p38 MAPK, and JNK-1/2/3. Cotreatment of chondrocytes with S1P diminished the IL-1β–induced phosphorylation of p38 MAPK (Figure 6A), whereas no impact on the phosphorylation of ERK-1/2, JNK-1/2/3, or NF-κB p65 was observed.

To test whether the inhibition of p38 MAPK phosphorylation was responsible for the diminished expression of iNOS in the chondrocytes, we used PD169316, a pharmacologic inhibitor of p38 MAPK. Blockade of the activation of p38 MAPK (with PD169316), but not blockade of ERK-1/2 (with U0126) or JNK (with SP600125), resulted in significant inhibition of the IL-1β–induced expression of iNOS (*P* = 0.001) (Figure 6B). Furthermore, we examined whether signaling through S1P_2_ was responsible for the diminished activation of p38 MAPK in response to S1P. Cotreatment of chondrocytes with the S1P_2_ inhibitor JTE-013 prevented the S1P-mediated reduction in p38 MAPK phosphorylation (Figure 6C). From these experiments, we conclude that S1P reduces IL-1β–induced p38 MAPK phosphorylation and iNOS expression by signaling through S1P_2_-dependent pathways.

## DISCUSSION

High levels of S1P are present in the synovial fluid of OA patients, amounting to 0.8–3.5 μ*M*, which clearly exceeds normal plasma concentrations (22,25). Given that our findings revealed the expression of S1P receptors in cartilage chondrocytes, S1P signaling seems to be of biologic relevance in OA cartilage. As different S1P receptor subtypes exert different and sometimes opposing effects, the impact of S1P on cellular signaling depends on the receptor subtypes available on the cell surface (20). We identified S1P_2_ as the most abundant S1P receptor in human OA chondrocytes, ex vivo and in vitro. In the presence of the functional S1P_2_ receptor, S1P diminished the cytokine-induced expression of iNOS and altered IL-1β signaling by abrogation of p38 MAPK phosphorylation.

S1P_2_ is a unique G protein–coupled receptor that preferentially signals via the G_α12/13_ pathway (30). Downstream signaling involves activation of the Rho/ROCK/PTEN pathway, as well as the NF-κB and MAPK pathways (31–33). We showed that inhibition of iNOS expression by S1P is not dependent on ROCK or PTEN in chondrocytes. Furthermore, S1P did not have an impact on IL-1β–induced phosphorylation of NF-κB p65. In contrast, phosphorylation of p38 MAPK was inhibited by S1P cotreatment. This result was surprising, since, in prior studies, S1P activated ERK-1/2 and p38 MAPK in chondrocytes (21,22). However, those studies were performed in the absence of IL-1β. Furthermore, activation of ERK-1/2 and p38 MAPK was inhibited by PTX, indicating that G_αi_ and S1P_1_ could have some involvement. In the current study, PTX did not fully reverse the effect of S1P.

These findings suggest that S1P triggers 2 alternate pathways that have an impact on p38 MAPK. One pathway may involve signaling through S1P_1_ and G_αi_, which enhances the phosphorylation of p38 MAPK. A second pathway, involving S1P_2_ independent of G_αi_, inhibits the phosphorylation of p38 MAPK. Similarly, opposing functions have been described for S1P_1_ and S1P_2_ in other cells (34). Our data imply that the inhibitory pathway prevails in the presence of IL-1β and may represent a physiologic mechanism limiting excessive iNOS expression, thereby preventing cellular damage by NO. We further demonstrate that induction of iNOS expression by IL-1β is dependent on p38 MAPK, but not on JNK or ERK. This finding is consistent with prior studies in chondrocytes, the results of which have indicated that activation of both p38 MAPK and NF-κB are required for IL-1 to fully induce the expression of iNOS (35–37).

Chondrocytes from damaged cartilage areas had lower expression of S1P_2_, and the relative number of chondrocytes positive for S1P_2_, compared to those positive for S1P_1_, decreased with increasing cartilage damage. As a result, chondrocytes in damaged cartilage are less likely to bind S1P via the S1P_2_ receptor, and thus S1P in chondrocytes derived from damaged cartilage lacks the inhibitory effect on iNOS and ADAMTS-4 expression. Consistent with this observation, we found that in chondrocytes from damaged cartilage, expression of iNOS and ADAMTS-4 was not reduced within 3 hours of cotreatment with S1P. Only after 12 hours of culture in the presence of IL-1β, chondrocytes from the damaged cartilage of some OA patients eventually showed an increase in the expression of S1P_2_ and were then able to respond to S1P to the same extent as that in chondrocytes from intact cartilage after 3 hours. The increase in S1P_2_ expression correlated with responsiveness to the inhibitory effect of S1P. This observation implies a negative feedback loop by which IL-1β increases S1P_2_ expression in chondrocytes from damaged cartilage and, thus, limits the release of NO and ADAMTS-4. Furthermore, this feedback loop was dysfunctional in the chondrocytes from damaged cartilage of some OA patients.

In the current study, we investigated the role of S1P and its receptors in different stages of OA. We did not, however, examine cartilage from healthy individuals. Given our findings in OA cartilage, future studies exploring the physiologic role of S1P in healthy cartilage should be performed.

We have herein demonstrated that S1P interferes with IL-1β signaling in human chondrocytes. S1P diminishes IL-1β–induced iNOS expression. Functionally, we described a novel signaling pathway linking S1P_2_, p38 MAPK, and iNOS expression. These results add new aspects to our understanding of human cartilage biology and may have therapeutic implications in light of the novel class of S1P agonist drugs currently being developed (38).
